# MicroRNA-27a-3p enhances the inflammatory phenotype of Juvenile Idiopathic Arthritis fibroblast-like synoviocytes

**DOI:** 10.1186/s12969-023-00833-8

**Published:** 2023-06-06

**Authors:** Claire H. Bullock, Sarah M. McAlpine, Sarah E. Roberts, Beata Derfalvi

**Affiliations:** 1 Dalhousie Medical School, Faculty of Medicine, Dalhousie University, Halifax, Canada; 2 Division of Immunology, Dept. of Pediatrics, Dalhousie University, IWK Health, 8 East Research 5850/5980 University Ave Halifax, NS B3K 6R8 Halifax, Canada

**Keywords:** Fibroblast-like synoviocytes, Inflammation, Juvenile idiopathic arthritis, microRNA, Synovial fluid

## Abstract

**Background:**

Juvenile Idiopathic Arthritis (JIA) is the most prevalent chronic pediatric rheumatic disorder. In joints of JIA patients, aggressive phenotypic changes in fibroblast-like synoviocytes (FLS) of the synovial lining play a key role in inflammation. MicroRNAs are dysregulated in rheumatoid arthritis and JIA, including miR-27a-3p. However, it is not understood if miR-27a-3p, enriched in JIA synovial fluid (SF) and leukocytes, alters FLS function.

**Methods:**

Primary JIA FLS cells were transfected with a miR-27a-3p mimic or a negative control microRNA (miR-NC) and stimulated with pooled JIA SF or inflammatory cytokines. Viability and apoptosis were analyzed by flow cytometry. Proliferation was evaluated using a ^3^H-thymidine incorporation assay. Cytokine production was assessed by qPCR and ELISA. Expression of TGF-β pathway genes was determined using a qPCR array.

**Results:**

MiR-27a-3p was constitutively expressed in FLS. Overexpression of miR-27a-3p caused increased interleukin-8 secretion in resting FLS, and interleukin-6 was elevated in SF-activated FLS compared to miR-NC. Furthermore, stimulation with pro-inflammatory cytokines augmented FLS proliferation in miR-27a-3p-transfected FLS relative to miR-NC. Expression of multiple TGF-β pathway genes was modulated by overexpression of miR-27a-3p.

**Conclusions:**

MiR-27a-3p significantly contributes to FLS proliferation and cytokine production, making it a potential candidate for epigenetic therapy that targets FLS in arthritis.

## Background

Juvenile Idiopathic Arthritis (JIA) is the most common rheumatic disorder in pediatric patients however, its etiology remains largely unknown [1, 2]. While the diagnostic criteria, prognosis, and treatments vary among the JIA subtypes, all JIA patients experience joint inflammation, reduced mobility and chronic pain resulting from unregulated inflammation to either exogenous or endogenous antigens [1, 3]. Furthermore, untreated or poorly managed JIA can lead to functional disability caused by local growth disturbances, or to a number of variable extra-articular manifestations, depending on the specific JIA subtype. Chronic anterior uveitis in oligoarticular or RF-negative polyarticular JIA can cause blindness, or Macrophage Activation Syndrome in the systemic subtype can be life-threatening [1, 4].

Current therapies for JIA include non-specific anti-inflammatory medications such as non-steroidal anti-inflammatory drugs and glucocorticoids, disease modifying antirheumatic drugs, and expensive biologics [5]. Though these therapies have dramatically changed the therapeutic landscape, up to 50% of JIA patients still have active disease in adulthood [6] and consistent therapy is required to avoid consequential damage and permanent loss of function [7]. Consequently, the identification of novel, targeted therapies could improve outcomes for patients with JIA, Rheumatoid Arthritis (RA) and potentially other inflammatory diseases.

In JIA, inflammatory cells infiltrate the synovium and cause hyperplasia, while producing pro-inflammatory cytokines including tumor necrosis factor (TNF)-α, interleukin (IL)-1β, and IL-6, leading to bone and cartilage damage due to deposition of immune complexes [5]. Additionally, fibroblast-like synoviocytes (FLS) in the synovial membrane produce inflammatory cytokines and matrix metalloproteinases which degrade the cartilage [8]. In adult RA, aggressively proliferating FLS have other phenotypic changes such as loss of contact inhibition, causing increased proliferation, decreased apoptosis and expression of oncogenes [8]. The mechanisms underlying pro-inflammatory changes in FLS during arthritic inflammation are not completely understood.

​ MicroRNAs are small, non-coding RNA molecules that regulate protein translation at the mRNA level, modulating many cellular pathways including differentiation, proliferation, and apoptosis [9]. The dysregulation of microRNAs has been demonstrated in various inflammatory and autoimmune diseases, including RA [9]. In the inflamed joint, microRNAs are elevated or decreased in RA patients compared to osteoarthritis patients [10]. Additionally, several plasma microRNAs have been shown to be predictive of remission after treatment in RA patients, including miR-27a-3p, which is higher in responders at baseline but decreases after treatment with adalimumab and methotrexate [11]. In FLS from RA patients, inflammatory cytokines such as IL-1β and TNF-α can induce microRNA production [12], and microRNAs such as miR-155 and miR-27a can induce or inhibit the proliferation and invasion of FLS [13–16].

While knowledge of microRNA dysregulation in RA can serve as a base for microRNA research in JIA, JIA has distinct pathophysiology from RA [17]. Several studies have shown that multiple microRNAs are dysregulated in the blood of JIA patients [18–21]. Additionally, we previously showed using highly sensitive, absolute quantitative droplet-digital PCR that the levels of various microRNAs were significantly different in JIA synovial fluid (SF) relative to JIA blood, including miR-27a-3p [21]. Furthermore, miR-27a-3p was elevated in the fluid and leukocytes of the joint compared to white blood cells and plasma [21]. Nziza et al. described a microRNA signature in SF that distinguishes JIA from septic arthritis [22]. Despite these advances in our knowledge, no studies have examined mechanistically the role of microRNAs in JIA FLS.

Here, we investigated the effects of miR-27a-3p overexpression on FLS from JIA patients. We hypothesized that miR-27a-3p promotes FLS proliferation and cytokine production, which could exacerbate inflammation in the arthritic joint.

## Methods

### Study subjects

Whole SF collected in acid citrate dextrose anticoagulant was obtained from eight JIA patients (Table 1) undergoing SF aspiration for acute joint inflammation at Dalhousie University/IWK Health Center (Halifax, Nova Scotia, Canada). All patients had oligoarticular JIA according to the International League of Association for Rheumatology classification [23]. Written informed consent was obtained from the patients’ legal guardian(s). This study was performed according to the Declaration of Helsinki and was approved by the IWK Research and Ethics Board (#1005378).

**Table 1:** Demographic and clinical manifestations of JIA patients at enrollment

	JIA Patients
Number of patients	8
Age at consent, years - mean (SD)	8.5 (5.5)
Sex – number (%)	
Male	3 (37%)
Female	5 (63%)
ILAR classification – n (%)	
Oligoarticular arthritis	7 (88%)
Extended oligoarticular arthritis	1 (12%)
Symptom duration, months – mean (SD)	19.3 (27.2)
ANA^+^ – number (%)	7 (83%)
Active joint count – mean (SD)	1.7 (1.0)
Medications - %	
Treatment naive	5 (63%)
NSAIDs only	3 (37%)

*ILAR *International League of Association for Rheumatology, *ANA *Antinuclear antibodies, *NSAIDs *Non-steroidal anti-inflammatory drugs

### FLS cell culture

SF samples collected in acid citrate dextrose tubes were centrifuged to pellet cells, and FLS were cultured as previously described [24, 25] and cryopreserved. The cell-free SF was stored at -80 °C. FLS culture homogeneity was confirmed by flow cytometry using anti-CD90-FITC (clone 5E10, BioLegend, San Diego, USA), anti-CD14-PE (clone 61D3, eBioscience, San Diego, USA), and fixable viability dye APC-eFluor780 (ThermoFisher Scientific, Mississauga, CA) to exclude dead cells. The samples were acquired on a BD LSRFortessa flow cytometer (BD Biosciences, Mississauga, CA). Highly pure FLS were used at passage 4–6 for experiments. Twenty-four hours prior to stimulation or transfection, culture media was replaced with AIM-V serum-free media (Gibco, Waltham, USA) to avoid serum microRNA contamination.

### FLS transfection with miR-27a-3p

Monolayers of FLS (75% confluent) were transfected with the fluorescein amidite-labelled miRCURY LNA miRNA mimic miR-27a-3p (5’UUCACAGUGGCUAAGUUCCGC) or a non-targeting negative control Cel-39-3p mimic (miR-NC; 5’UCACCGGGUGUAAAUCAGCUUG), both from Qiagen (Germantown, USA). Lipofectamine RNAiMAX Transfection Reagent (Invitrogen, Waltham, USA) was used according to the manufacturer protocol, with the following modification: the mimic was prepared in Opti-MEM® I Reduced Serum Medium (Gibco, Waltham, USA), using 1 µL of RNAiMAX for every 500 µL of cell culture, and a final mimic concentration of 10 nM. An equal volume of Opti-MEM® was added to the no-transfection control wells.

### FLS activation

Twenty-four hours post-transfection the cells were stimulated for 24 h with a cocktail of 10 ng/mL each of IL-6, IL-1β, and TNF-α (Peprotech), or ¼ of the culture media volume was replaced with cell-free SF pooled from five treatment naïve oligoarticular JIA patients. After activation, the cells were lysed with QIAzol (Qiagen) or were trypsinized and analyzed by flow cytometry as described below. Culture supernatants were stored at -80 °C.

### Evaluation of microRNA and cytokine gene expression by reverse-transcriptase-quantitative PCR (qPCR)

RNA was extracted from FLS lysates using the miRNeasy Mini Kit as per manufacturer’s instructions (Qiagen), including an on-column DNase digest, and RNA was eluted in 40µL of molecular-grade ultrapure water (Invitrogen). For microRNA targets, cDNA was synthesized from 5 µL of RNA using the miScript II RT Kit following manufacturer’s instructions (Qiagen). For mRNA targets, cDNA was generated from 5 µL of cDNA using the iScript cDNA Synthesis kit (Bio-Rad). All cDNA preparations were diluted 1/10 before freezing at -20 °C.

Reactions to detect microRNA by qPCR (20 µL total) consisted of 1x Sso Advanced Universal SYBR Green Supermix (Bio-Rad, Hercules, USA), 0.25 µM forward (Table 2) and Universal Reverse (Qiagen) primers, 5 µL of cDNA template, and molecular-grade ultrapure water (20 µL total reaction volume). Reactions to quantify cytokine gene expression were the same as above and included forward and reverse primers (Table 2), but no Universal Reverse primer was added. The reactions were run on the CFX Connect Real-Time System thermocycler (Bio-Rad) using the following settings: activation at 95 °C for 30 s, 40 cycles of [denaturation at 95 °C for 10 s, annealing for 15 s at the indicated temperature (Table 2), and extension at 70 °C for 20 s]. Melt curve analysis was included post-cycling. All data were analyzed using CFX Maestro software (Bio-Rad). MiR-27a-3p was normalized using the geometric mean of the reference genes miR-93-5p and miR-191-5p. GAPDH and HPRT1 were used as reference genes for cytokine gene expression.

**Table 2:** Primers and annealing temperatures

Target	Primer Sequence or Commercial Product	Supplier	Annealing Temperature (°C)
miR-27a-3p	TTCACAGTGGCTAAGTTCCGC	Invitrogen	55
miR-93-5p	GCAAAGTGCTGTTCGTG	Invitrogen	55
miR-191-5p	CGCGCAACGGAATCCCA	Invitrogen	55
CCL2	PrimePCR assay	Bio-Rad	60
CXCL8	PrimePCR assay	Bio-Rad	53
GAPDH	PrimePCR assay	Bio-Rad	60
HPRT1	PrimePCR assay	Bio-Rad	60
IL6	RT^2^ qPCR Primer Assay	Qiagen	60
IL17A	PrimePCR assay	Bio-Rad	53
MMP3	Sense: 5’AGTCTTCCAATCCTACTGTTGCT Antisense: 5’TCCCCGTCACCTCCAATCC	Invitrogen	53

*CCL2 *C-C motif chemokine ligand 2, *CXCL8 *C-X-C motif chemokine ligand 8, *GAPDH *Glyceraldehyde 3-phosphate dehydrogenase, *HPRT1 *Hypoxanthine phosphoribosyltransferase 1, *MMP3 *Matrix metalloproteinase 3, *TNFA *Tumor necrosis factor alpha

### Enzyme-linked immunosorbent assay (ELISA)

IL-6, IL-8, and CCL2 protein levels were assessed in FLS culture supernatants by ELISA according to manufacture’s protocol (eBioscience). Absorbances were measured on a SpectraMax 190 and data were analyzed using SoftMax Pro v4.3 (both from Molecular Devices, San Jose, USA).

### Assessment of proliferation and apoptosis

A ^3^H-thymidine incorporation assay was used to determine the effect of transfection and stimulation on FLS proliferation as outlined by Long et al. [26]. Briefly, each treatment condition was performed in triplicate in 96-well plates. FLS were transfected and activated as described above. Eighteen hours before the end of the activation, 1 µCi of ^3^H-thymidine (ARC Inc., St. Louis, USA) was added to each well. The samples were measured on a TriCarb 4810TR scintillation counter (Perkin-Elmer). To assess apoptosis, FLS were stained with Annexin-V-PE (BD Biosciences) and analyzed by flow cytometry.

### TGF-β pathway qPCR array

The expression of multiple TGF-β pathway genes was evaluated in FLS using cDNA prepared as described above and TGF-β Receptor Signalling H384 Primer assay plates, run according to manufacturer instructions (Bio-Rad). Each target was normalized to the reference genes GAPDH, HPRT1 and TBP. The normalized data were expressed as follows: fold increase in expression with miR-27a-3p compared to miR-NC with and without SF stimulation.

### MicroRNA target and pathway analysis

Potential targets and pathways associated with miR-27a-3p were evaluated using DIANA-mirPath v.3 with Tarbase and KEGG analysis [27].

### Data analysis and statistics

Hypothesis-driven comparisons were performed using a Student’s or ratio paired t-test as appropriate (normal distribution) or Wilcoxon matched-pairs signed rank test (not normally distributed). GraphPad Prism was used for statistical analysis and graph preparation. *P* values < 0.05 were considered significant.

## Results

### Characteristics of the study subjects

The study included a cohort of eight oligoarticular JIA patients who were treatment naïve or on non-steroidal anti-inflammatory drugs (NSAIDs) only; female:male ratio 5:3, mean age 8.5 ± 5.5 years, mean duration of symptoms 19.3 ± 27.2 months. Table 1 shows the demographics and clinical characteristics of the patients.

### Expression of miR-27a-3p and functionality of FLS after transfection

**Fig. 1:**
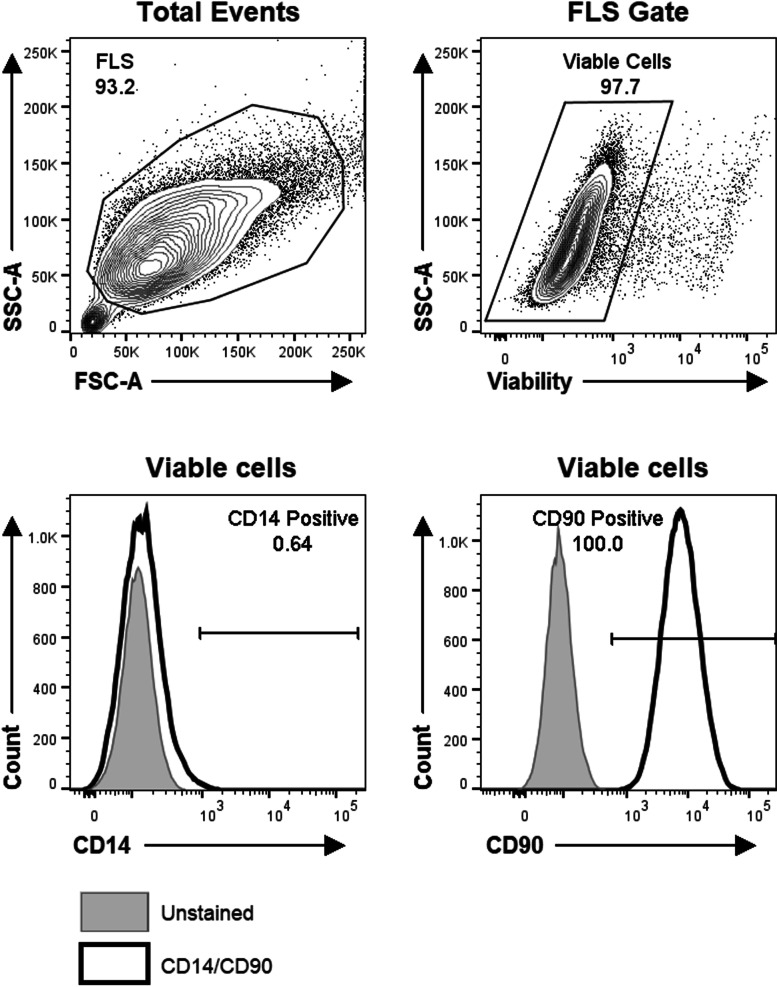
Primary oligoarticular JIA FLS are highly homogeneous. FLS grown from JIA synovial fluid samples were evaluated by immunofluorescence and flow cytometry. FLS were first gated based on light scatter properties, then dead cells were excluded. FLS stained with anti-CD14 and anti-CD90 (red histograms) were compared with the background fluorescence of pooled, unstained FLS (blue histograms). Data are representative of FLS cell cultures from six different JIA patients

**Fig. 2:**
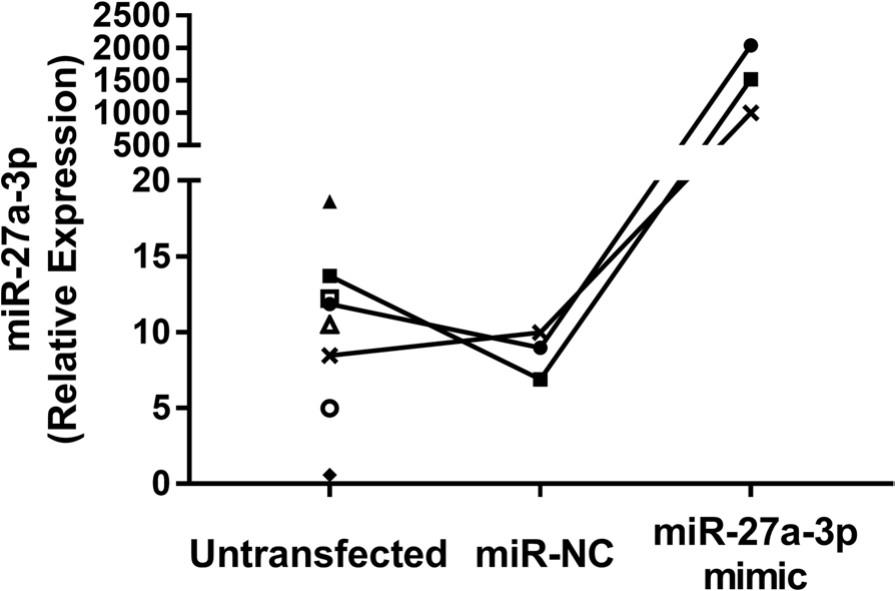
MiR-27a-3p is expressed constitutively in FLS and is highly induced upon transfection with miR-27a-3p mimic. FLS were cultured from oligoarticular JIA patients. Quantification of miR-27a-3p was performed by RT-qPCR in FLS without transfection, and 48 h after transfection with miR-NC or a miR-27a-3p mimic. The data were normalized to reference genes miR-93-5p and miR-191-5p. The graph shows individual, matched FLS cultures plotted with different symbols (*n* = 3–8)

The cultured FLS cells were 100% CD90-positive and 99.4% CD14-negative, and were therefore homogeneous primary oligoarticular FLS (Fig. 1). MiR-27a-3p was constitutively expressed in all FLS in the absence of stimulation or transfection (Fig. 2). Transfection efficiency measured in selected FLS transfected with miR-27a-3p mimic showed an average fold-increase in miR-27a-3p of 148 ± 21 (*n* = 3 out of 8 primary cells) compared to untransfected FLS (Fig. 2), with viability of > 96% (*n* = 8). To examine whether transfection affected FLS functionality, mRNA gene expression and protein production of multiple pro-inflammatory mediators was quantified in unstimulated and SF-stimulated FLS before and after transfection (Table 3). IL6 and MMP3 mRNA were consistently upregulated in FLS in response to SF regardless of transfection status, demonstrating that transfection does not inhibit FLS in this capacity. When comparing mock-transfected (miR-NC) to untransfected FLS, no differences were observed in IL6 or MMP3 gene expression in either resting or SF-stimulated cells, indicating that the transfection procedure does not interfere with cellular responsiveness.

**Table 3:** FLS pro-inflammatory potential remained intact after miR-27a-3p transfection

Readout		Untransfected (*n* = 5)			miR-NC (*n* = 8)			miR-27a-3p (*n* = 8)		
		Medium	SF	Fold Change	Medium	SF	Fold Change	Medium	SF	Fold Change
mRNA	IL6	13.0 ± 5.4	*226.1 ± 62.4	26.9 ± 9.6	16.1 ± 3.1	*370.5 ± 130.9	21.5 ± 5.0	30.1 ± 10.7	**421.5 ± 119.8	20.7 ± 6.9
	IL8	n.d.	n.d.	n.d.	5.4 ± 1.2	**16.8 ± 2.5	4.4 ± 1.1	7.1 ± 1.8	*70.0 ± 50.5	14.6 ± 11.3
	CCL2	n.d.	n.d.	n.d.	3.2 ± 0.3	**35.0 ± 7.3	10.8 ± 1.9	6.4 ± 2.8	**41.9 ± 9.2	9.1 ± 1.4
	MMP3	7.0 ± 3.0	89.1 ± 42.3	30.7 ± 20.3	7.3 ± 1.6	*61.2 ± 18.5	9.5 ± 3.1	12.4 ± 6.1	**3094.1 ± 3039.1	63.4 ± 55.0
Protein	IL-6	n.d.	n.d.	n.d.	41.9 ± 8.1	***632.5 ± 38.9	79.1 ± 64.0	49.6 ± 10.7	****654.6 ± 35.0	70.1 ± 56.5
	IL-8	n.d.	n.d.	n.d.	13.7 ± 2.4	***255.1 ± 32.2	21.7 ± 4.1	20.3 ± 1.7	**251.5 ± 38.5	13.3 ± 2.8
	CCL2	n.d.	n.d.	n.d.	972.7 ± 220.6	***5790.8 ± 681.6	6.7 ± 0.7	942.1 ± 198.4	***5570.3 ± 818.1	6.4 ± 0.7

Data are presented as relative expression values for mRNA and pg/mL for protein. Fold changes were calculated as SF compared to medium. Data are presented as mean ± SEM. *p<0.05, **p<0.01, ***p<0.001, ****p<0.0001, when comparing medium to SF within cells of the same transfection status. SF, synovial fluid

### Effect of miR-27a-3p overexpression on FLS cytokine gene expression and protein secretion

**Fig. 3:**
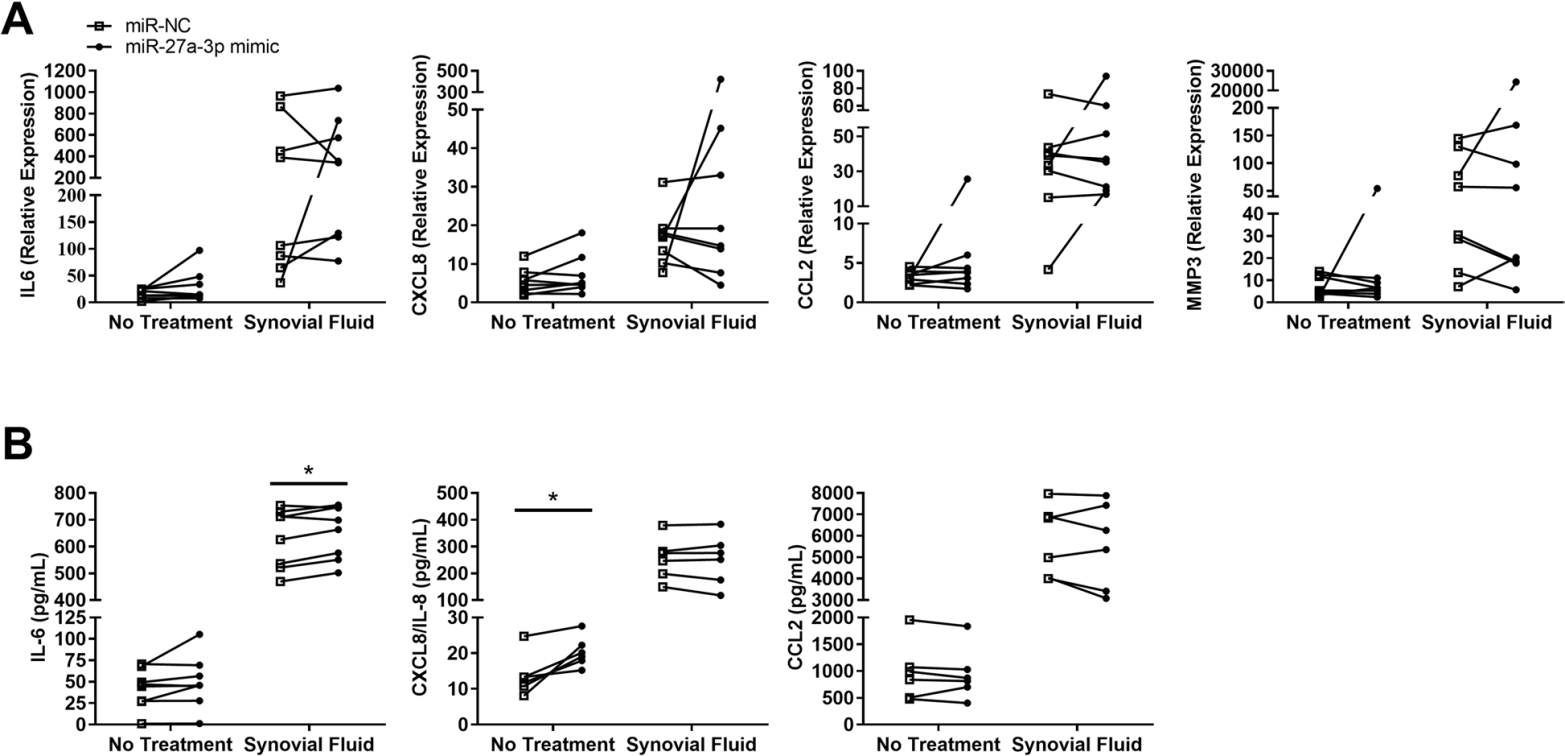
Transfection with miR-27a-3p enhances FLS cytokine secretion. FLS were transfected with either miR-27a-3p mimic or miR-NC, then stimulated with pooled JIA synovial fluid (SF), or were left unstimulated. **A** Expression of the indicated cytokine genes was quantified using RT-qPCR, normalized to reference genes HPRT1 and GAPDH. Graphs show individual paired samples (*n* = 8). **B** Secretion of IL-6, CXCL8/IL-8, and CCL2 protein in culture supernatants was quantified by ELISA. Graphs show individual paired samples (*n* = 6–8). *p < 0.05

FLS overexpressing miR-27a-3p exhibited no significant changes in mediator gene expression in the absence of stimulation (Fig. 3A). SF stimulation induced higher overall expression of IL6, IL8, CCL2 and MMP3 mRNA, however no significant differences were observed in FLS overexpressing miR-27a-3p compared to miR-NC (Fig. 3A). Resting FLS also expressed mRNA for IL17A (32.0 ± 11.6 relative expression, *n* = 8). However, FLS overexpressing miR-27a-3p did not differ significantly in the expression of IL17A when in a resting state (0.84 ± 0.21 fold change, *p* = 0.641) or when stimulated with SF (1.23 ± 0.29 fold change, *p* = 0.547).

At the protein level, there was a significant increase in IL-8 secretion by resting FLS transfected with miR-27a-3p compared with miR-NC (Fig. 3B). Furthermore, SF-stimulated FLS showed less marked but significantly elevated secretion of IL-6. Transfection did not affect CCL2 protein production.

### Effect of miR-27a-3p on FLS proliferation and apoptosis

**Fig. 4:**
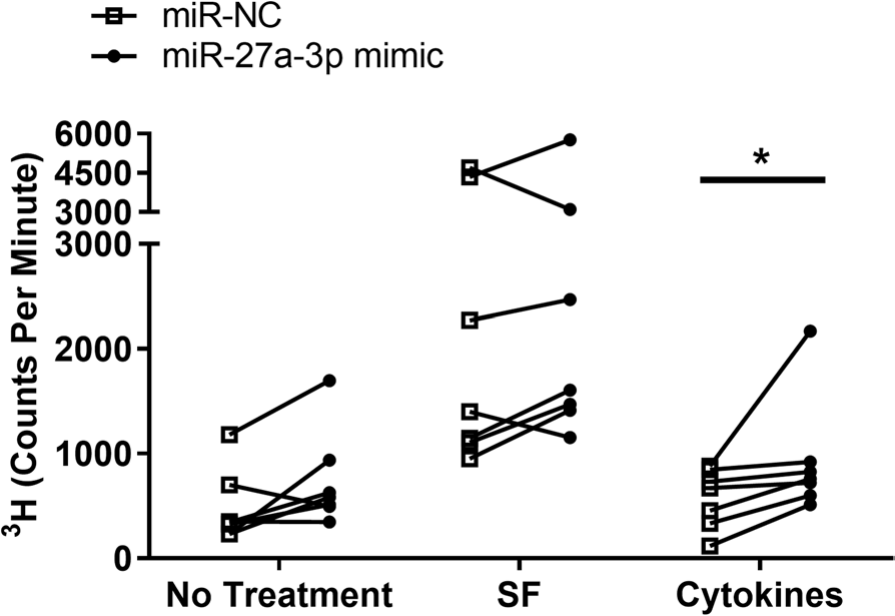
Transfection with miR-27a-3p enhances FLS proliferation. FLS were transfected with either miR-27a-3p mimic or miR-NC, then stimulated with pooled JIA synovial fluid (SF) or the cytokine combination IL-1β, IL-6 and TNF (cytokines). Unstimulated FLS were used as a no treatment control. Cell proliferation was determined by ^3^H-thymidine incorporation assay. Graph shows ^3^H incorporation in counts per minute in individual paired FLS cultures (*n* = 7). *p < 0.05

Transfection of FLS with miR-27a-3p induced higher proliferation rates in both resting and SF-stimulated cells, although this was not statistically significant (Fig. 4). However, when FLS were stimulated with the pro-inflammatory cytokines IL-1β, IL-6 and TNF, proliferation was significantly higher in FLS overexpressing miR-27a-3p compared to miR-NC (Fig. 4).

Annexin V staining showed no difference between rates of apoptosis in FLS transfected with the miR-27a mimic relative to miR-NC when they were unstimulated (1.22 ± 0.23 fold change, *p* = 0.641) or stimulated with SF (1.50 ± 0.48 fold change, *p* = 0.844) or pro-inflammatory cytokines (1.12 ± 0.17 fold change, *p* = 0.547).

### Effect of miR-27a-3p on the TGF-β pathway in FLS

**Fig. 5:**
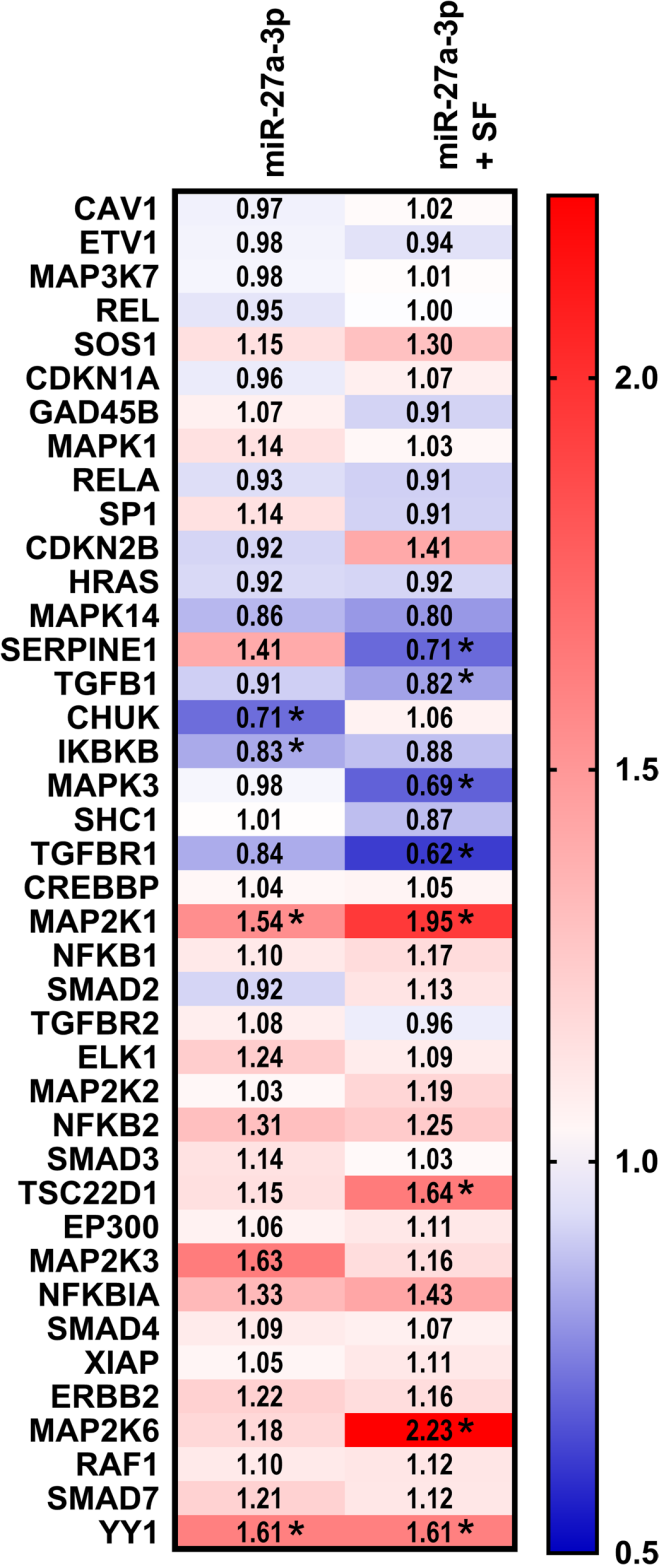
Overexpression of miR-27a-3p modulates the expression of genes in the TGF-β pathway in FLS. FLS were transfected with miR-27a-3p mimic or miR-NC and cultured with or without pooled JIA synovial fluid (SF). Gene expression was quantified by qPCR array and the data were normalized to three reference genes as described in Methods. The heat map illustrates the fold-change in mRNA expression in FLS cells overexpressing miR-27a-3p relative to miR-NC without stimulation (left) or with SF stimulation (right) (*n* = 6). *p < 0.05

MiR-27a-3p has been shown to target Follistatin-like 1, a TGF-β1-inducible protein, altering RA-FLS migration, invasion and cytokine production [13]. Additionally, pathway analysis showed strong association of multiple miR-27a-3p targets with the TGF-β pathway (*p* = 4.1 × 10^− 5^). To further investigate the effects of miR-27a-3p overexpression on the TGF-β pathway in FLS, a qPCR array was performed (*n* = 6). Figure 5 shows that miR-27a-3p significantly decreased the expression of CHUK and IKBKB mRNA and increased the expression of MAP2K1 and YY1 mRNA in resting FLS compared to FLS transfected with miR-NC. After SF stimulation, miR-27a-3p mimic-transfected FLS had decreased expression of MAPK14, SERPINE1, MAPK3 and TGFBR1 mRNA, whereas expression of TSC22D1, MAP2K1, YY1 and MAP2K6 was increased, compared with miR-NC-transfected FLS stimulated with SF.

## Discussion

MicroRNAs are dysregulated in numerous inflammatory diseases including RA, yet little is known of their role in JIA, especially their expression in JIA FLS. We have shown previously that miR-27a-3p is enriched in the inflamed joint, both in SF and SF leukocytes, relative to blood [21], and here we demonstrated that miR-27a-3p is constitutively expressed in JIA FLS cells (Fig. 2). Therefore, this result indicates that FLS may contribute to the miR-27a-3p present in SF, in addition to other known sources of miR-27a-3p such as leukocytes [21] and chondrocytes [28]. Additionally, overexpression of miR-27a-3p by transfection induced higher rates of proliferation in FLS (Fig. 4). It is possible that a positive feedback loop occurs, where resting FLS produce miR-27a-3p which in turn induces proliferation, thereby generating a larger FLS population in the joint which can subsequently produce more miR-27a-3p. Further studies should investigate which pro- and anti-inflammatory stimuli affect miR-27a-3p production in FLS.

MiR-27a-3p overexpression induced significantly higher IL-8 protein production in resting FLS (Fig. 3). Since microRNAs regulate gene expression directly by reducing protein translation, we hypothesize that miR-27a-3p alters IL-8 expression upstream, indirectly. IL-8 is a potent chemoattractant for neutrophils [29], and it has been shown that neutrophils in JIA patients are more mature and have an activated phenotype, particularly in SF [30]. Therefore, miR-27a-3p may underlie the mechanism of neutrophil recruitment via FLS production of IL-8. We also observed significantly higher cytokine-induced cell proliferation and SF-induced IL-6 secretion in FLS (Fig. 3). IL-6 is a well-known pro-inflammatory cytokine, a common target of biologic therapy, with multiple functions including antibody production and hematopoiesis, and it plays a detrimental role in arthritis by promoting osteoclast formation and bone resorption [31]. Therefore, these findings support a pro-inflammatory role for miR-27a-3p in JIA FLS. Similarly to us, inflammation signature and protein synthesis in FLS were studied by others after a range of 18–30 h of stimulation with pro-inflammatory cytokines [13, 32–34]. However, there could be differences in cytokine-specific kinetics in mRNA and protein expression that were not addressed in this study. An earlier time point may have been needed to observe similar differences in cytokine gene expression. In a single RA study upregulation of miR-27a-3p had the opposite effect, suppressing proliferation and inflammatory response (IL-6, IL-1 and TNF production) of FLS, supposedly through silencing of TLR5. However, the experimental design was different in that tissue derived FLS cells were studied with modest miRNA transfection efficiency and cell proliferation was analyzed with a colorimetric assay [32].

The transfection procedure did not affect baseline cytokine gene expression and production, and FLS maintained their biological responsiveness to SF activation. Similar Lipofectamine-delivered overexpression of non-coding RNA is used with high transfection efficiency in a broad spectrum of cell types (including FLS) with good cell viability and functionality maintained [35–38]. It should be noted that a limitation of this study was the small sample size. The findings should be confirmed with a separate validation cohort.

Notably, cytokine levels in supernatants were much higher when FLS were stimulated with SF (Fig. 3). SF contains many pro-inflammatory mediators that activate FLS, including cytokines, chemokines and lipid-derived mediators [39, 40]. Some of the protein present in supernatants may have been contained in the SF itself. However, FLS clearly upregulated gene expression of IL-6, CXCL8 and CCL2 in response to SF, strongly suggesting that FLS secreted these proteins during activation. Transfection induced a less pronounced difference in cytokine levels compared to SF stimulation. Titrating the concentration of SF for FLS activation may reveal more obvious differences in FLS transfected with miR-27a-3p. Additionally, activated, rather than resting FLS, may be more susceptible to epigenetic modulation caused by overexpression of miR-27a-3p due to stimulation-induced changes of multiple targeted genes.

There was variability in FLS cytokine responses even though all FLS were derived from oligoarticular JIA patients who were treatment-naïve or on NSAIDs only. JIA is classified based on clinical presentation and number of joints affected, however, our data suggests that there are individual differences at the cellular level that affect FLS behaviour and potentially response to treatment even in this clinically homogenous group.

An additional approach to further explore the mechanistic effects of miR-27a-3p modulation would be to silence this microRNA using synthetic antisense oligonucleotides (antagomirs), however current strategies may not change the expression levels or function of the target microRNA due to low binding affinity and biostability (easy degradation by nucleases) of anti-miR constructs [41].

Our research indicated that miR-27a-3p plays an important role in regulating mRNA expression of several TGF-β pathway genes (Fig. 5) that influence cell proliferation and adhesion (MAP2K1, MAPK3, SERPINE1), apoptosis (CHUK), and inflammation via NF-κB signalling or other pathways (MAP2K6, MAPK14, CHUK, IKBKB [42]. Collectively, our results suggest that miR-27a-3p may play a role in mediating JIA FLS aggressive pro-inflammatory and proliferative phenotypic changes, which may contribute to joint inflammation at the pannus-cartilage junction. These findings are distinct from RA, where miR-27a-3p overexpression decreased proliferation and cytokine production, and increased apoptosis in resting FLS [13, 32]. These disparate findings may be explained by key differences in experimental design and/or in the JIA and RA pathophysiology. Assessing the level of proteins such as TGF-β receptor 1 and MAP2K6 in SF-stimulated FLS transfected with miR-27a-3p would strengthen these findings.

Our findings on the pathomechanism of oligo JIA can not be generalized or extrapolated to the other subtypes of JIA. JIA subtypes show a variety of characteristics, biomarkers, and systemic features due to different pathophysiological mechanisms, caused by HLA genetic pre-disposition, effector cells and cytokines involved in the aetiopathogenesis [43, 44]. We consider it a strength of our study that we focused exclusively on the persistent oligoarticular JIA, which is the least heterogeneous subtype. Our findings in oligoarticular JIA FLS characteristics are probably not applicable across all the JIA subtypes and might be very different for example in ERA or SJIA. There is minimal literature on overlapping biological characteristics of FLS in rheumatoid arthritis and JIA and there is very limited comparable work done on FLS in JIA subtypes. Comparative transcriptome analysis and high-throughput single-cell RNA-sequencing revealed biologically relevant differences in gene expression in FLS cells distinguishing JIA subtypes, and support a critical role for FLS in pathogenesis [45]. Heterogeneity described even within one defined JIA subtype complicates these comparisons even further. Especially the chondrocyte-like cells subpopulation within the FLS cells (which increase with disease severity) have unique genetic fingerprints that distinguish between JIA subtypes [46]. Further similar experiments using FLS cells from other JIA subtypes or RA would demonstrate whether FLS cells behave similarly to oligo JIA.

## Conclusions

This is the first demonstration that miR-27a-3p, which is enriched in inflamed JIA joints, augments JIA FLS biological functions, including cell proliferation and pro-inflammatory cytokine production. These results suggest that the actions of miR-27a-3p may allow ongoing joint inflammation to persist, rather than resolve, in JIA patients. By identifying microRNAs that modulate disease activity, we aim to better understand the disease process. Future research should focus on the utility of microRNA as predictors of treatment response and as potential therapeutic targets in the JIA patient population.

## Data Availability

The datasets used and analysed during the current study are available from the corresponding authors on reasonable request.

## Abbreviations

CCL: C-C motif chemokine ligand
CHUK: Conserved helix-loop-helix ubiquitous kinase
CXCL: C-X-C motif chemokine ligand
ELISA: Enzyme-linked immunosorbent assay
FLS: Fibroblast-like-synoviocytes
GAPDH: Glyceraldehyde 3-phosphate dehydrogenase
HPRT1: Hypoxanthine phosphoribosyltransferase 1
IKBKB: Inhibitor of nuclear factor kappa B kinase subunit beta
IL: Interleukin
JIA: Juvenile Idiopathic Arthritis
MAPK: Mitogen-activated protein kinase
MAP2K: Mitogen-activated protein kinase kinase
miR-NC: negative control microRNA
MMP: Matrix metalloproteinase
NSAIDs: Non-steroidal anti-inflammatory drugs
RA: Rheumatoid Arthritis
qPCR: Quantitative PCR
RT: Reverse transcriptase
SERPINE1: Serpin family E member 1
SF: Synovial fluid
TBP: TATA-box binding protein
TGF: βTransforming growth factor beta
TGFBR1: Transforming growth factor beta receptor 1
TNF-α: Tumour necrosis factor alpha
TSC22D1: TSC22 domain family member 1
YY1: Yin yang 1
